# Treatment preference and recruitment to pediatric RCTs: A systematic review

**DOI:** 10.1016/j.conctc.2019.100335

**Published:** 2019-02-19

**Authors:** L. Beasant, A. Brigden, R.M. Parslow, H. Apperley, T. Keep, A. Northam, C. Wray, H. King, R. Langdon, N. Mills, B. Young, E. Crawley

**Affiliations:** a Centre for Academic Child Health, Bristol Medical School, University of Bristol, UK; b Department of Academic Paediatrics, Royal Alexandra Children's Hospital, Brighton and Sussex University Hospitals, UK; c NHS Greater Glasgow and Clyde, UK; d Department of Primary Care and Public Health, Royal Sussex County Hospital, Brighton and Sussex Medical School, UK; e Population Health Sciences, Bristol Medical School, University of Bristol, UK; f Bristol Royal Hospital for Children, University Hospitals Bristol NHS Trust, UK; g Institute of Psychology, Health and Society, University of Liverpool, UK

**Keywords:** Randomised controlled trial, Recruitment, Pediatric, Parent, Treatment preference

## Abstract

**Background:**

Recruitment to pediatric randomised controlled trials (RCTs) can be a challenge, with ethical issues surrounding assent and consent. Pediatric RCTs frequently recruit from a smaller pool of patients making adequate recruitment difficult. One factor which influences recruitment and retention in pediatric trials is patient and parent preferences for treatment.

**Purpose:**

To systematically review pediatric RCTs reporting treatment preference.

**Methods:**

Database searches included: MEDLINE, CINAHL, EMBASE, and COCHRANE.

Qualitative or quantitative papers were eligible if they reported: pediatric population, (0–17 years) recruited to an RCT and reported treatment preference for all or some of the participants/parents in any clinical area. Data extraction included: Number of eligible participants consenting to randomisation arms, number of eligible patients not randomised because of treatment preference, and any further information reported on preferences (e.g., if parent preference was different from child).

**Results:**

Fifty-two studies were included. The number of eligible families declining participation in an RCT because of preference for treatment varied widely (between 2 and 70%) in feasibility, conventional and preference trial designs. Some families consented to trial involvement despite having preferences for a specific treatment. Data relating to ‘participant flow and recruitment’ was not always reported consistently, therefore numbers who were lost to follow-up or withdrew due to preference could not be extracted.

**Conclusions:**

Families often have treatment preferences which may affect trial recruitment. Whilst children appear to hold treatment preferences, this is rarely reported. Further investigation is needed to understand the reasons for preference and the impact preference has on RCT recruitment, retention and outcome.

## 1 Introduction

Successful recruitment and retention is crucial in randomised controlled trials (RCT) research [[1], [2], [3]]. Recruitment problems can delay or prevent trial completion [[4], [5], [6], [7], [8], [9], [10], [11]], and post-randomisation drop-out can lead to the loss of statistical power to measure differences between treatment arms [4,7,9,12,13]. Exploration of recruitment and retention issues in trials is extensive. Factors highlighted as important during the design and implementation phases of RCTs include: trial design, incentives, patient characteristics, support for recruiters, and patient and recruiter preferences for treatment [1,2,4,[14], [15], [16], [17], [18]].

If patients have a preference for treatment offered in an RCT they may decline randomisation to access treatment outside the trial. The external validity of an RCT may be compromised if patients with treatment preferences decline to participate, and bias is possible if uneven numbers of participants drop-out or cross-over between treatment arms [19,20]. Preferences can also affect adherence to treatment arms in RCTs where blinding to trial interventions is not possible [21,22]. Trials recruiting adult patients have reported treatment preference as a barrier to recruitment [[23], [24], [25]], but there it is a lack of evidence in relation to the ways in which preferences for trial interventions might affect recruitment and retention in pediatric trial settings [18].

Systematic reviews investigating the effects of treatment preference in RCTs have largely focused on trials recruiting adult patients [26,27]. A systematic review published in 2005 investigated the effects of participants' and professionals' preferences on recruitment, retention, and treatment outcomes. This review extracted data from 34 RCTs, but only four of the included trials had recruited pediatric-patients. Preferences were found not to significantly affect trial validity, but preferences did influence patients’ willingness to participate [26]. The second systematic review published in 2008 focused on musculoskeletal trials, extracting data from 18 RCTs none of which recruited pediatric patients [27]. This review investigated the effect of preference on attrition and outcomes but did not investigate the effect of treatment preference on recruitment. It found that patient preferences for treatment were associated with treatment effects.

We cannot assume treatment preferences will have the same impact on recruitment to pediatric trials as has been shown in adult trials. Pediatric trials involve the combined preferences of parent(s), patient and recruiting clinicians, in addition to a more complex consent process [28,29]. There will also be variation in the extent to which young people participate in decision-making and the recruitment process, depending on the nature and severity of their illness [[30], [31], [32], [33], [34], [35]]. The purpose of this systematic review was to identify pediatric RCTs where treatment preferences are reported, and describe the impact of preference on recruitment and retention.

## 2 Methods

A review protocol was developed and registered with PROSPERO: https://www.crd.york.ac.uk/PROSPERO/display_record.asp?ID=CRD42015015942. The review protocol also included methodology relating to the syntheses of qualitative data extracted from papers identified via this systematic literature search, which will be submitted for publication separately [36].

### 2.1 Study eligibility and inclusion criteria

Scoping exercises were used to define and refine relevant search terms using the PICOC model: Population, Intervention, Comparison, Outcomes and Context [37]. Qualitative sub-studies embedded in RCTs or quantitative primary and secondary outcome papers were eligible for inclusion if they reported RCTs recruiting new-borns, children and young people aged 0–17 years to an RCT, in any clinical area. Eligible papers were also required to report treatment preferences for all or some of the participants/parents. Database searches were limited to 1950–2014 inclusive.

### 2.2 Search strategy

A search strategy was developed with guidance from University of Bristol data specialists (NIHR/CLAHRC West and Cochrane Collaboration group), the search strategy can be found in Supplemental Information, Appendix A. Database searches of MEDLINE, CINAHL, EMBASE, and COCHRANE were carried out. Searches of relevant reference lists, databases containing registered clinical trials (https://www.ukctg.nihr.ac.uk/https://clinicaltrials.gov/ct2/home http://www.anzctr.org.au/TrialSearch.aspx) and work not published in peer-reviewed journals (http://proquest.umi.com/login) were carried out.

Authors were contacted to establish whether full RCT results had been published, two provided copies of their papers [38,39] and three confirmed that they had not [[40], [41], [42], [43]].

### 2.3 Screening and data extraction

Each title and abstract was screened independently for inclusion by two researchers using the systematic review platform Covidence [44]. Discrepancies were documented, discussed and resolved in regular meetings by reviewers and a senior member of the study management team (EC) to ensure eligibility criteria were understood and screening queries resolved consistently. At the full text review stage papers were read in chronological order by two researchers (LB and AB, HK, RL or RP). Author(s) extracted relevant numeric data and/or descriptive reports of treatment preference into an Excel template (see Supplemental Information, Appendix B).

## 3 Results

### 3.1 Summary of included studies

**Fig. 1:**
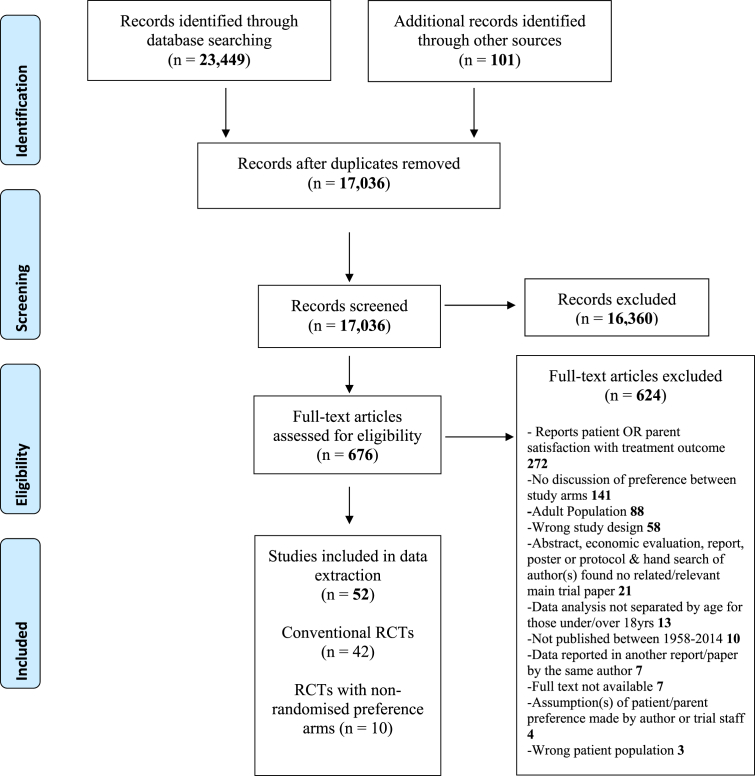
PRISMA [107] Systematic search of literature reporting treatment preference in pediatric RCTs.

Database searches retrieved 23,449 papers, and additional searches yielded 101 papers. After deduplication, title and abstract screening was carried out on 17,036 papers, and 676 were read in full, with 52 papers eventually included in analyses (see Fig. 1). Table 1 describes the papers included in the systematic review. Twenty-seven papers reported data from RCTs conducted in the UK [28,29,[45], [46], [47], [48], [49], [50], [51], [52], [53], [54], [55], [56], [57], [58], [59]] and Europe [38,40,41,[60], [61], [62], [63], [64], [65], [66]], 16 from RCTs conducted in the USA and Canada [39,42,43,[67], [68], [69], [70], [71], [72], [73], [74], [75], [76], [77], [78], [79]], seven in countries outside of North America and Europe [30,[80], [81], [82], [83], [84], [85]], and two papers reported RCTs collecting data internationally [86,87]. Most papers were published from the year 2000 onwards (n = 42). Of the 52 papers included, 24 described ‘primary’ trial outcomes and 28 were ‘secondary’ papers which explored patient/parent experience of trial involvement, or reasons for declining, consenting, and recruitment. Searches were carried out to locate primary trial papers for secondary papers included in the review and 18 were located [[88], [89], [90], [91], [92], [93], [94], [95], [96], [97], [98], [99], [100], [101], [102], [103], [104], [105]]. It was not possible to find all the primary trial papers because some secondary papers didn't explicitly use identifiable trial names or registration numbers. Of the 52 papers, seven reported findings from multiple trials [[28], [29], [30],^39^,^48^,^52^,^70^], and two were abstracts from poster presentations [40,41]. Forty-two of the papers reported ‘conventional’ RCTs [[28], [29], [30],[38], [39], [40], [41], [42], [43],[45], [46], [47],^49^,^50^,^52^,[54], [55], [56], [57], [58], [59], [60],^62^,^64^,^65^,[67], [68], [69], [70], [71],^74^,^75^,[77], [78], [79], [80], [81], [82], [83],[85], [86], [87]], two of which were in the feasibility or pilot stages [46,80]. Eight papers described RCTs with parallel ‘preference’ arms at trial outset [51,53,61,66,72,73,84,106], and two introduced preference arms due to slow recruitment [48,76].

**Table 1:** Included studies (n = 52).

Conventional RCTs (n = 42)			
Author	Paper type (primary or secondary paper^a^)	Participant age	Aim
Allen 2013 [80]	Primary (Feasibility)	13–17yrs	Assessed feasibility of recruiting young women into an RCT of caseload midwifery.
Allmark 2006 [45]	Secondary Primary paper Azzopardi 2009 [90]	≥36wks	Compared intensive care plus total-body cooling for 72 h with intensive care without cooling among term infants with asphyxial encephalopathy.
Banks 2012 [46]	Primary (Pilot)	5–16yrs	Assessed feasibility of carrying out a fully powered RCT comparing; care of childhood obesity intervention (COCO) and a primary care clinic intervention (PCC).
Barratt 2013 [81]	Secondary Primary paper Wake 2009 [104]	5–10yrs	In-depth understanding of why families chose not to participate in a community-based study on childhood obesity.
Bauchner 1996 [67]	Primary	3mth-6yrs	Do parents prefer antibiotic administration for treatment of acute otitis media by a single intramuscular (IM) injection or standard oral therapy for 10 days.
Blickman 2013 [68]	Primary	1–12yrs	Assessed the impact of a Certified Child Life Specialist (CCLS) on parent satisfaction, staff satisfaction, child satisfaction, and parent and staff perceptions of child pain and distress in a pediatric imaging department.
Byrne-Davis 2010 [47]	Secondary Primary paper Vora 2013 [103]	2–11yrs	Examined how recruitment looked to an observer and how it felt to parents, (of children with low-risk acute lymphoblastic leukemia) to identify how doctors' communication could promote or inhibit optimal recruitment.
Caldwell 2003 [30]	Secondary (Multiple RCTs)	Not stated	Explored parents' attitudes to children's participation in trials, identifying factors that influenced decision making and perceived risks and benefits. RCTs included oncology and renal: interventions not defined.
Carvalho 2013 [82]	Secondary Primary paper Moreira 2013 [99]	<3yrs	The understanding and perceptions of mothers regarding the informed consent and randomisation processes linked to an RCT that compared behavior management techniques for pediatric dental sedation.
Chappuy 2014 [38]	Secondary	Children - age not stated	Parental and child understanding of RCT participation (Acute lymphoblastic leukemia FRALLE 2000A protocol) and evaluations of the readability of written documents provided.
Duncan 2004 [69]	Primary	11mths-12yrs	Effectiveness of osteopathic manipulation, acupuncture or wait list control as a 6-month therapeutic adjunct for children with spastic cerebral palsy.
Eiser 2005 [49]	Secondary Primary paper Mitchell 2005 [98]	4–16yrs	Mothers' (of children newly diagnosed with Acute Lymphoblastic Leukemia: ALL) views regarding consent to randomised controlled trials.
Forsander 1995 [60]	Primary	12–15yrs	Evaluation of family attitudes in relation to the two 3wk care systems for diabetes management: early discharge from ward to training apartment and treatment on a ward in pediatric clinic.
Glogowska 2001 [50]	Secondary Primary paper Glogowska 2000 [94]	3–4yrs	Reported attitudes of parents whose child took part in a speech and language therapy RCT comparing immediate treatment and watchful waiting.
Harth 1990 [83]	Secondary Primary paper Van Asperen 1992 [101]	6mths-3yrs	Double-blind, placebo-controlled trial of ketotifen, a new and unlicensed (for Australia) oral asthma drug.
Hissink Muller 2011 [40]	Secondary (poster presentation) Primary paper Hissink Muller 2017 [96]	Children - age not stated	Comparison of three treatment strategies, and feedback relating to treatment preferences among parents of patients with recent onset juvenile idiopathic arthritis.
Hissink Muller 2012 [41]	Secondary (poster presentation) Primary paper Hissink Muller 2017 [96]	12–18yrs	Comparison of three treatment strategies, and feedback relating to equipoise among parents and patients with recent onset juvenile idiopathic arthritis.
Johnson 2007 [42]	Secondary	10–18yrs (and adults)	Assessed participant and parent experiences in the parenteral insulin arm of the Diabetes Prevention Trial (DPT-Type 1).
Johnson 2009 [43]	Secondary	10–18yrs (and adults)	Assessed the experiences of participants and parents of children in the oral insulin study of the Diabetes Prevention Trial (DPT-Type 1).
Jollye 2009 [52]	Secondary (Multiple RCTs)	Neonates	Explored the thoughts and feelings of parents in their decision-making process, in either choosing or declining to participate in neonatal RCTs.
Levi 2000 [70]	Secondary (Multiple RCTs)	2–18yrs	Retrospective parent perceptions of communication of their child's cancer diagnosis and the informed consent process.
Miner 2007 [71]	Primary	6mth-17yrs	To determine if nebulized fentanyl is a feasible alternative to IV fentanyl for the treatment of acute pain in children presenting to the emergency department (ED) with painful conditions.
Payne 2004 [54]	Secondary	3–12yrs	Views and preferences for anesthetic related issues important to parents (and adults) who took part in a prospective RCT.
(PENTA) Paediatric European Network for Treatment of AIDS 1999 [86]	Secondary (double-blind)	Children - age not stated	Described parents' experience of their child being enrolled in a HIV infection RCT, including the degree to which it interfered with life, and their feelings about use of deferred (placebo) and immediate antiretroviral treatment.
Rovers 2000 [62]	Primary	16-24mths	The effectiveness of ventilation tubes on the language development in infants with persistent otitis media with effusion (OME) compared to watchful waiting (WW).
Sammons 2007 [55]	Secondary Primary paper Atkinson 2007 [89]	6mth-16yrs	Parental views on the informed consent process, information provided, reasons for taking part and willingness to participate in future research. Compared motives of British and European parents.
Sandler 2014 [56]z	Primary	12–18yrs	Effectiveness of 3 methods of orthodontic anchorage supplementation, reporting orthodontists' and patients' values.
Sartain 2002 [57]	Primary	6wks-12yrs	Assessed the clinical effectiveness of a pediatric hospital at home service compared to conventional hospital care.
Schuttelaar 2010 [64]	Primary	≤16yrs	Compared the level of care from nurse practitioners with care delivered by dermatologists.
Sederberg-olsen 1998 [65]	Secondary (double blind) Primary paper Balle 1998 [91]	1–10yrs	Evaluated the efficacy of amoxicillin-clavulanate and penicillin-V in the treatment of secretory otitis media (SOM).
Shilling 2011 [28]	Secondary (Multiple RCTs) MASCOT: funding extension application rejected & trial closed prematurely [97] MENDS [88] POPs [still recruiting] TIPIT [108]	MASCOT: 6–15yrs MENDS: 3–15yrs POP: 4–18yrs TIPIT: < 28wks	Identify strategies to improve recruitment and trial conduct, by comparing practitioners' and parents' accounts of the invitation to enter a child into clinical trials.
Snowdon 1997 [58]	Secondary Primary paper UK Collaborative ECMO Trial Group [95]	Neonates	Exploration of parental reactions to random allocation of treatment in a neonatal RCT comparing two methods of life support; conventional management (CM) and extracorporeal membrane oxygenation (ECMO). Recruitment was stopped early, because data showed a clear advantage with ECMO.
Spandorfer 2005 [74]	Primary Loss of clinical equipoise and declining accrual rates led to trial termination.	8wk-3yrs	Compare oral rehydration therapy (ORT) and intravenous fluid therapy (IVF) in the treatment of viral gastroenteritis.
Sureshkumar 2012 [85]	Secondary Primary paper Craig 2009 [92]	<18yrs	To identify modifiable and unmodifiable factors associated with parental consent to a trial investigating long-term, low-dose antibiotics in preventing recurrent urinary tract infection.
Tercyak 1998 [75]	Secondary Primary paper Diabetes Control Complications Trial Research Group [93]	11–18yrs	Identify reasons/characteristics of adolescents who refuse or consent to participate in an RCT of intensive therapy (IT) for insulin-dependent diabetes mellitus.
Willey 2005 [59]	Primary	4–16yrs	Efficacy of oral or rectal route administered analgesia for post-operative pain.
Williams 2013 [77]	Primary	2–17yrs	Compared cast versus splint for distal radial buckle fractures in children in terms of parental and patient satisfaction, convenience and preference.
WoodgateZ 2010 [39]	Secondary (Multiple RCTs)	6mth-15yrs	In-depth understanding of Canadian parents' participation in decisions about childhood cancer clinical trials.
Woolfall 2013 [29]	Secondary (Multiple RCTs) MASCOT [97] funding extension application rejected & trial closed prematurely. MENDS [88] POPs [still recruiting] TIPIT [108]	MASCOT: 6–15yrs MENDS: 3–15yrs POP: 4–18yrs TIPIT: < 28wks	Explored how a parent's understanding of a trial might be associated with the way that the trial was explained during the discussion with a practitioner.
Wright 2005 [87]	Primary Recruitment was expected to take 3yrs but took 6yrs.	4–10yrs	Investigated early application hip spica compared with external fixation in pediatric femoral fractures. Recruitment was expected to take 3yrs but took 6yrs.
Wynn 2010 [78]	Secondary Primary paper Wang 2011 [105]	<18mths	In response to slow recruitment study coordinators evaluated factors that affected enrollment and accrual.
Young 2006 [79]	Secondary	7–17yrs	Reported results of two studies of social phobia, assessing the extent to which parental reluctance toward medication resulted in pre-treatment attrition in; behavioural, fluoxetine and placebo groups.
**RCTs with non-randomised preference arms (n** = **10)**			
Cunningham 2011 [48]	Secondary Trial 1: preference arm added and trial terminated early due to inadequate sample size.	Adolescents (age not stated)	Reported two RCTs, both terminated early due to inadequate sample size. Trial 1: Multi-center Orthodontic RCT which compared two different methods of treating a specific type of malocclusion in adolescents. (Trial 2: RCT, no preference data).
Gowers 2010 [51]	Primary	12–18yrs	Compared the clinical effectiveness of inpatient against outpatient treatment and of generalist against specialist management.
Lock 2010 [53]	Primary Trial extended from 5 to 7yrs to increase patient recruitment.	4–15yrs	An embedded qualitative study informed the development of the RCT, it explored patient/parent(s) preferences for different treatment options in patients with recurrent sore throats who had recently been referred to ENT clinic. Extended from 5 to 7yrs to increase patient recruitment.
Mattila 2007 [61]	Primary	≤2yrs	Assessed adenoidectomy in connection with tympanostomy compared with tympanostomy only in preventing otitis media in children.
Paradise 1984 [72]	Primary	3–15yrs	Assessed the efficacy of tonsillectomy and adenoidectomy.
Paradise 1990 [73]	Primary	1–15yrs	Assessed the efficacy of adenoidectomy, comparing surgical and non-surgical management, with equivalent non-randomised preference arms.
Reddihough 1998 [84]	Primary	12-36mths	Compared conductive education (CE) program with equivalent intensity traditional neurodevelopmental programs of rehabilitation for young children with Cerebral Palsy.
Rovers 2001 [106]	Primary	9–12mths	Compared ventilation tubes (VT) and watchful waiting (WW) in the management of patients with otitis media with effusion. The generaliszability of randomised patients with eligible non-randomised patients was studied via preference arms.
Weinstein 2013 [76]	Primary Preference arms added after 3yrs of recruitment.	10–15yrs	The effectiveness of bracing, compared with observation in preventing progression of the curve to 50° or more in idiopathic scoliosis patients, with equivalent non-randomised preference arms.
Van Wijk 2014 [66]	Secondary Primary paper Van Wijk 2014 [102]	4.5–6.5mths	Primary: Effectiveness of helmet therapy for positional skull deformation compared with the natural course of the condition Secondary: Assess parents' decision for helmet therapy in infants with skull deformation.

a Primary papers were defined as those reporting primary RCT outcome(s). Secondary papers were those reporting embedded/related studies (e.g. qualitative) describing patient/parent experience of trial involvement, reasons for decline, consenting and recruitment.

### 3.2 Impact of treatment preference on recruitment – conventional RCTs

Table 2 describes data on preference from all included papers. Seventeen papers reported the number of eligible families declining participation because of a preference for treatment, this ranged from 2 to 50% in conventional trials [49,[54], [55], [56], [57],^64^,^65^,^68^,^69^,^74^,^75^,^78^,^79^,^83^,[85], [86], [87]], and 4–70% in the two pilot/feasibility phase trials [46,80]. Eleven RCTs reported the preferences of families who opted for trial participation [38,[40], [41], [42], [43],^49^,[58], [59], [60],^67^,^77^], these treatment preferences were either expressed at enrolment or after randomisation. Five trials reported withdrawal after randomisation [57,62,71,74,82]. Families either withdrew consent or refused their allocated intervention, but only one of these trials specifically attributed this to a preference for the alternate treatment arm [71].

**Table 2:** Number of eligible participants recruited to trial, and those not randomised due to treatment preference.

Conventional RCTs (n = 42)			
Author	Number of eligible participants consenting to randomisation arms	Number of eligible patients not randomised because of treatment preference n (%)	Is preference expressed by patients (in addition to parents)
Allen 2013	1 (10%) (Feasibility)	7 (70%)	Yes (only patient preference reported)
Allmark 2010	325 (81%)	Unclear, preference reported qualitatively [45] ‘30 declined’ ‘45 other reasons’ [90]	n/a neonates
Banks 2012	76 (50%) (Pilot)	6 (4%)	No
Barratt 2013	258 (33%)	Not reported. 9 (26%) of non-responders reported concern with being in either the intervention or control group, but only 37/305 non-responders replied to question.	No
Bauchner 1996	648 (total eligible not reported)	Not reported. Parents were asked their preference at enrollment and 551 (85%) of those randomised preferred single-dose therapy over standard therapy.	n/a children under 6yrs
Blickman 2013	142 (88%)	4 (2%)	Unclear (patients aged 4yrs + were asked to complete a standardised study instrument)
Byrne-Davis 2010	521 (71%) [103]	Not reported, preference reported qualitatively [47] 215 (29%) not randomly assigned; 97 refused, 7 had Down's syndrome, 4 because of toxic effects, 28 other reason, 79 unknown [103]	No
Caldwell 2003	Not reported (multiple trials)	Not reported, preference reported qualitatively.	Participant age not stated.
Carvalho 2013	Unclear 48 'recruited' [82] 44 (100%) 'randomised' [99]	Not reported, preference reported qualitatively [82] 3 (7%) parents refused allocated interventions post-randomisation in x 2 trial arms [99]	No
Chappuy 2014	Not reported	Not reported. Some Parents felt that standard treatment was the best arm for their child because it was less risky	Participant age not stated.
Duncan 2004	50 different participants randomised. Total eligible not reported.	8 (between 12 and 16%)	No
Eiser 2005	1621 (90%) [98]	181 (10%) declined randomisation (opted for PRED; 165 DEXA; 16) [98] Preference reported qualitatively, 16 (32%) ‘agreed reluctantly to randomisation’ [49].	No
Forsander 1995	38 (93%)	Not reported Immediately after randomisation 3 families in the control arm reported that they would have preferred the family therapeutic care arm.	No
Glogowska 2001	159 (69%) [94]	Not reported, preference reported qualitatively [50] Declined trial in total 70 (31%) [94]	n/a children under 4yrs
Harth 1990	72 (55%)	40 (30%) families declined because of ‘concern about side effects of the new drug’ (ketotifen) 60 declined in total.	n/a children under 3yrs
Hissink Muller 2011	Not reported	Not reported. 41% participating parents reported a preference for therapy with methotrexate and etanercept and 6% had hoped against assignment to this group. Primary aversion was highest (25%) in the prednisone group [40]. Declined trial n = 38 (29%) [96].	No
Hissink Muller 2012	Not reported	Not reported. 65% participating parents reported a preference for therapy with etanercept. 5 parents and 2 patients participated in the study to access treatment with etanercept, as initial treatment was not possible nor reimbursed in daily practice.	Yes
Johnson 2007	Not reported	Not reported. Participating families stated: Close monitoring arm - 27% parents and 70% participants were glad to be in that arm. 74% parents and 35% participants sometimes wished they had been assigned the intervention arm. Intervention arm - 53% parents and 21% participants were glad to be in that arm. 25% parents and 47% participants sometimes wished they had been assigned the closely monitored arm.	Yes
Johnson 2009	Not reported	Not reported. Participating families were blinded to treatment but were asked which treatment arm they would have preferred. 60% parents and 53% participants chose the capsule condition. 8% parents and 21% participants chose the no intervention condition. Very few participants and parents (3%) chose the insulin injection condition.	Yes
Jollye 2009	Not reported, multiple trials.	Not reported, preference reported qualitatively.	n/a neonates
Levi 2000	Not reported, multiple trials.	Unclear. 3 (13.6%) stated they declined participation because they felt more comfortable with a “tried and true” method.	No
Miner 2007	41 (82%)	Unclear. Declined randomised 9 (18%) reasons not reported. After allocation 4 (10%) parents requested that their child receive nebulized fentanyl rather than the assigned IV fentanyl.	No
Payne 2004	Unclear Calculated as; 322 (69%) of eligible patients. Paper reports recruitment rate of 75%	59 (50%) ‘Around half of the eligible participants who refused to participate did so because there was a 50% chance of the child being randomised to the inhalational induction arm’.	No
(PENTA) Paediatric European Network for Treatment of AIDS 1999	197	4 (3%) parents stated explicitly that they were concerned with the use of placebo.	No
Rovers 2000	187	Not reported. 19 (10%) parents withdrew consent straight after randomisation (15 in ventilation tubes arm and 4 in watchful waiting arm). 10 (5%) children in the watchful waiting arm were treated with ventilation tubes.	n/a children under 2yrs
Sammons 2007	Unclear 245 'randomised’ [55] 252 (85%) ‘randomised’ [89]	25 (9%) declining families stated they wanted a specific treatment (IV; 20 or oral; 5) [55] 43 (15%) declined to take part; n = 6 (2%) excluded post randomisation reasons: 4 withdrawn by parents/2 by clinician (no further detail provided) [89].	No
Sandler 2014	78 (87%)	7 (8%) Three did not want to wear headgear for anchorage, three did not want the Nance button palatal arches, but only one patient did not want to take part because he or she was unhappy at “the thought of temporary anchorage devices”.	No
Sartain 2002	399 (86%)	10 (2%) 7 families withdrew from ‘hospital care’ arm because they wanted the ‘hospital at home’ arm	Yes
Schuttelaar 2010	160	4 (2%) Preferred only dermatologist (n = 2), preferred only nurse practitioner (n = 2).	No
Sederberg-olsen 1998	429	120 (10%) parents insisted that the child had grommet insertion performed at the time of randomisation.	No
Shilling 2011	MASCOT: 63 [97] MENDS: 146 (84%) [88] POP: [still recruiting] TIPIT: 153 (57%) [108]	Unclear, preference reported qualitatively. MASCOT Assessed for eligibility (n = 898), Not registered (n = 732), Excluded (n = 103) [97]. MMENDS 27 (16%) assessed for eligibility but not randomised: ‘declined 11’ ‘other 16’ [88]. TIPIT 57 (21%) assessed for eligibility but not randomised: ‘refused’ [105].	Yes
Snowdon 1997	185 (79%) [95]	Unclear. ‘majority of parents had a keen preference for ECMO treatment arm’. Preference reported qualitatively [58]. 48 (21%) were registered but not randomised; 14 died, 19 improved and 15 parents refused trial participation [95].	n/a neonates
Spandorfer 2005	73	24 (7%) A further 3 parents refused participation after randomisation to oral rehydration therapy before starting treatment.	n/a children under 3yrs
Sureshkumar 2012	412 (37%) [85]	214 (19%) Prefer antibiotics 71/Prefer no antibiotics 143 [85]. Primary paper reports patients excluded because ‘participation refused by parent’ 1935 [92]	No
Tercyak 1998	56	2 (5%)	Yes (only patient preference reported)
Willey 2005	31	Not reported. 19/31 patients completed a preference questionnaire/10 (43%) preference for oral, 2 (9%) for suppositories, 7 (30%) no preference/preference for oral more pronounced among girls 5 (83%).	Yes
Williams 2013	94	Not reported. A significantly larger percentage of parents and patients in the cast group reported that they would not choose the same method of immobilization again at all time points (baseline, days; 1, 3, 7, 21 after injury).	No
Woodgate 2010	Not reported (multiple trials)	Not reported, preference reported qualitatively.	n/a neonates
Woolfall 2013	MASCOT: 63 [97] MENDS: 146 (84%) [88] POP: [still recruiting] TIPIT: 153 (57%) [108]	Unclear, preference reported qualitatively. MASCOT Assessed for eligibility (n = 898), Not registered (n = 732), Excluded (n = 103) [97]. MMENDS 27 (16%) assessed for eligibility but not randomised: ‘declined 11’ ‘other 16’ [88]. TIPIT 57 (21%) assessed for eligibility but not randomised; ‘refused’ [105].	No
Wright 2005	108 (46%)	41 (33%)	No
Wynn 2010	234 (29%)	2% unwilling to take placebo.	n/a children under 2yrs
Young 2006	Not reported.	125 ‘Reluctance toward medication treatment accounted for 44.7% of study refusals and was disproportionately common among ethnic minority families’.	No
RCTs with non-randomised preference arms (n = 10)			
Cunningham 2011	Not reported. (multiple trials)	Not reported. A small number of patients who were eligible declined the trial as they had a treatment preference. These were patients allocated to both intervention groups, so one treatment option was not preferred to the other. Preference arms added.	Unclear
Gowers 2010	170 (68%)	28 (11%) Not randomised, patient preference.	Yes
Lock 2010	268 (26%)	286 (28%) declined any follow up, authors assumed that all had a patient preference. 461 (45%) opted for preference arms in cohort.	Only in qualitative sample. Authors did not attempt to differentiate between parent/child preferences in RCT/preference samples.
Mattila 2007	137 (45%)	169 (55%) opted for preference arms.	n/a children under 2yrs
Paradise 1984	91 (49%)	96 (51%) opted for preference arms.	No
Paradise 1990	99 (46%)	114 (54%) opted for preference arms.	No
Reddihough 1998	34 (49%)	32 (46%) declined randomisation.	n/a children under 3yrs
Rovers 2001	187 (48%)	133 (34%) opted for non-randomised cohort arms. 66 (17%) refused randomisation/follow up via cohort.	n/a children under 1yrs
Van Wijk 2014	84 (21%)	186 (46%) opted for preference arms.	n/a children under 1yrs
Weinstein 2013	155 (14%)	228 (21%) opted for preference arms. 297 (27%) declined all follow-up due to preference. 216 (20%) no to randomisation.	No

### 3.3 Impact of treatment preference – RCTs with non-randomised preference arms

Eight papers reported RCTs which used non-randomised ‘preference arms’ in addition to randomised treatment arms from the outset [51,53,61,63,72,73,84,102]. All of these trials reported the number of eligible families declining randomisation arms because of a preference for treatment, this ranged from 11 to 55%. One of these trials was extended by two years to increase recruitment to randomised trial arms [53]. Two additional trials introduced preference arms because families declined participation because of preferences for treatment [48,76].

### 3.4 Patient or parent preference

Nine papers explicitly reported the treatment preferences of patients, as well as their parents [28,[41], [42], [43],^53^,^57^,^59^,^75^,^80^]. Child/parental views on a preferred treatment arm differed on three occasions [28,42,53]. Twelve papers reported findings from trials involving children under the age of six years, so did not include information on preference from children [45,50,58,[61], [62], [63],^67^,^68^,^74^,^78^,^83^,^84^].

### 3.5 Clinician preferences for trial treatments

Most studies did not comment on why families held a treatment preference, but six papers reported different forms of clinician preference for a particular treatment which may have influenced patient preference [28,41,63,77,84,85]. Two trials stated that staff experienced discomfort with children's medication/intervention being selected by a process of randomisation [28,84], one highlighted that *‘consent was more likely when the recruiting physician was a member of the research team’* [85] and in another, a parent whose child was randomised to a splint treatment arm was told the day after randomisation by a clinician outside the RCT that *‘all buckle fractures need to be casted’* [77]. Finally, one trial reported that parents who refused randomisation did so because of; *‘a desire to have decisional control, and they trusted their physician's choice of treatment more than a computer's choice’* [109]. These findings suggest that recruiters and treating clinicians may be an important influence on parent and patient treatment preferences when families consider RCT participation.

## 4 Discussion

To our knowledge, this is the first systematic review that has specifically investigated whether treatment preference influences recruitment into pediatric trials. The review has shown that families often have preferences for treatment at recruitment, and some families consent to trial involvement despite having preferences for a specific treatment. The number of eligible families declining participation in an RCT because of preference for treatment varied widely: From 2 to 70% in feasibility RCTs, from 2 to 50% in conventional main RCTs, and from 11 to 55% in trials with preference arms. Declining accrual rates and a loss of clinical equipoise led to the closure of two trials [48,74], and two required extensions because of slow recruitment [53,87].

Several trials included in this systematic review introduced preference arms to improve recruitment. Patient preference trials (PPTs) and comprehensive cohort designs [110,111], (in which participants with a preference are offered their treatment of choice, and those without a preference have their treatment allocated randomly) offer the opportunity to investigate the effects of preference on recruitment, validity and treatment outcome [26,27,112]. Although this is one way of dealing with patients’ preferences for treatment, this design has a number of disadvantages. PPTs often require larger numbers of patients. In extending trial duration to meet recruitment targets for the randomised arms, they may reduce external validity and generalisability of results. Also, such designs do not necessarily improve informed consent [53,110,111,[113], [114], [115]].

A key strength of this review is that a large number of papers were screened for inclusion by two reviewers at all stages in the review process. This review was enriched by the inclusion of a wide range of papers, including data from papers reporting primary trial outcomes, and papers reporting qualitative findings on patient or parent experiences of trial involvement, and reasons for decline, consenting and recruitment. Limitations include the fact that seven papers reported findings from multiple trials in one paper [[28], [29], [30],^39^,^48^,^52^,^70^], and many of the papers reporting qualitative findings did not include full CONSORT flow diagrams, therefore data on those who were lost to follow-up or withdrew due to preference could not be extracted. The effect that treatment preference has on retention in pediatric trials requires further investigation. If trial acronyms or references were provided in secondary papers, we carried out a search for each related primary RCT outcome paper, but only 18/28 additional papers were located. Data relating to ‘participant flow and recruitment’ was not always reported consistently in primary RCT outcome papers. One paper reported that 76 participants were allocated to treatment arms, but only 68 then entered the RCT, presumably eight withdrew post-randomisation but reasons for this were not provided [46]. A lack of standardised detail in the reporting of recruitment and retention methodology in RCTs has also been highlighted previously in a systematic review of behavioural interventions recruiting dyads (adult patients and their support person) [116].

Parental reasons for strongly held treatment preferences include concerns about side effects and attitudes towards new ‘experimental’ or ‘placebo’ interventions [55,117,118]. Although altruism is often cited as a reason for RCT participation, there is also poor parental understanding of the process of randomisation and perceived personal benefit for their child [14,119]. In pediatric trials, parents and children are often both involved in receiving information about the trial and making a decision about whether to take part, with support from a recruiting clinician [120,121]. Our findings showed that parents' preferences are reported more frequently than children's preferences. Only nine papers reported child preference, even though the majority of included trials were conducted with children and young people who were old enough to assent to RCT involvement and express their views on treatment.

Children's preferences for treatment differed from parental views on three occasions [28,42,53]. Older children and teenagers have reported different views from their parents on the acceptability of treatment and participation in asthma research protocols [122]. This is not consistent with guidance suggesting young people's voices need to be more widely heard [35,123], or approaches to communication which aim to support personal autonomy instead of isolated ‘independence’ of choice in decision-making [124,125].

Although this systematic review was not seeking to report clinician preference for treatment in pediatric RCTs, a small number of studies did report that members of the recruiting/treating teams held preferences. The impact of clinician preference has been described as affecting pediatric trials [26,126]. In one trial 63% of parents said the doctors recruiting them had influenced their decision to participate [55]. Clinician preference has also been shown to influence recruitment in adult trials [4,[127], [128], [129]]. More research should be carried out to investigate the influence of recruiting professionals’ preferences for treatment on the decision-making process of families.

## 5 Conclusions

This systematic review shows that treatment preference can be a barrier to recruitment to pediatric RCTs. In some cases this can result in the need to change the design of the trial (introduction of preference arms), extend recruitment or result in trial closure. Further investigation is needed to understand the impact treatment preference has on retention, and on the outcomes under investigation in pediatric trials. Exploration of the reasons for parent and child preferences would also be beneficial to ensure that families are fully informed when making decisions about RCT participation.

## Conflicts of interest

The authors have no conflicts of interest relevant to this article to disclose.

## Financial disclosure

The authors have no financial relationships relevant to this article to disclose.

## Funding source

This work was undertaken with the support of the Medical Research Council (MRC) ConDuCT-II Hub (Collaboration and innovation for Difficult and Complex randomised controlled Trials In Invasive procedures - MR/K025643/1).

## Contributors’ Statement Page

All authors approved the final manuscript as submitted and agree to be accountable for all aspects of the work.
