# Supplementary material for: Treatment preference and recruitment to pediatric RCTs: A systematic review
Source: Contemp Clin Trials Commun. 2019 Feb 19;14:100335. doi: 10.1016/j.conctc.2019.100335 (PMC6430075; doi:10.1016/j.conctc.2019.100335)
Supplement: Multimedia component 1 [file mmc1.docx]

## **Supplemental Information, Appendix B**

| **Data extraction fields:**  Author  Primary outcome or secondary paper  Country  RCT type (full, feasibility, preference, comprehensive cohort)  RCT aim  Area of study & Description of interventions  Participant age (months/years)  Is preference expressed by patient/parent prior to randomisation  Is preference expressed by patients (in addition to parents)  Number of eligible participants consenting to randomisation arms  Number of eligible patients not randomised because of treatment preference n (%)  Post randomisation drop-out due to preference  Total: withdrawn/discontinued treatment/crossed over/lost to follow up  Further information reported on preference (included; preference of parent different from child, preference arms added, early trial closure, extension required, trial terminated early)  **Additional qualitative data extraction fields:**  Number of qualitative participants (parents/ participants)  Qualitative aim  Qualitative data collection method(s)  Qualitative approach & data analyses |
| --- |
