# Supplementary material for: Treatment preference and recruitment to pediatric RCTs: A systematic review
Source: Contemp Clin Trials Commun. 2019 Feb 19;14:100335. doi: 10.1016/j.conctc.2019.100335 (PMC6430075; doi:10.1016/j.conctc.2019.100335)
Supplement: Multimedia component 2 [file mmc2.docx]

## **Supplemental Information: Appendix A**

| Search strategy  Database: Medline 1950 to present  --------------------------------------------------------------------------------  1 adolescent/ or exp child/ (2516113)  2 minors/ (2367)  3 Pediatrics/ (42050)  4 (pediatri$ or paediatri$ or teenager$ or young person$ or young people).ti,ab. (245419)  5 (adolesc$ or boy$ or girl$ or child$ or juvenil$ or schoolchild$).ti,ab. (1213948)  6 or/1-5 (2862052)  7 (exp adult/ or adult.ti.) not (pediatri$ or paediatri$ or teenager$ or adolesc$ or young person$ or young people or boy$ or girl$ or child$ or juvenil$ or schoolchild$).ti,ab. (5590793)  8 6 not 7 (1740500)  --------------------------------------------------------------------------------  9 exp Clinical Trials as Topic/ (295653)  10 ((random* or crossover* or control* or cross-sectional or observational or longitudinal or clinical) adj4 (trial or trials or design or study or studies)).ti,ab. (1026721)  11 9 or 10 (1187255)  --------------------------------------------------------------------------------  12 ((patient$ or participant$ or parent$ or mother$ or father$ or child$ or carer$ or caregiver$ or care-giver$ or personal) adj3 (view or views or priorit$ or perception$ or prefer$ or belief$ or expectation$ or choice$ or perspective$ or satisfact$ or experience or experiences or opinion$ or concern or concerns or feeling$)).ti,ab. (189786)  13 exp patient satisfaction/ (65985)  14 professional-family relations/ or professional-patient relations/ or physician-patient relations/ or researcher-subject relations/ (97784)  15 12 or 13 or 14 (312317)  --------------------------------------------------------------------------------  16 8 and 11 and 15 (6445)  --------------------------------------------------------------------------------  17 letter/ (857062)  18 editorial/ (358231)  19 news/ (165244)  20 exp historical article/ (333331)  21 Anecdotes as topic/ (4682)  22 comment/ (581752)  23 case report/ (1739336)  24 (letter or comment$).ti. (91081)  25 or/17-24 (3403060)  --------------------------------------------------------------------------------  26 randomized controlled trial/ or Randomized Controlled Trials as Topic/ or random$.ti,ab. (848254)  27 25 not 26 (3371992)  --------------------------------------------------------------------------------  28 animals/ not humans/ (4004886)  29 exp Animals, Laboratory/ (764211)  30 exp Animal Experimentation/ (6725)  31 exp Models, Animal/ (447357)  32 exp rodentia/ (2802604)  33 (rat or rats or mouse or mice).ti. (1119422)  34 or/27-33 (7983465)  --------------------------------------------------------------------------------  35 16 not 34 (6323)  MEDLINE search carried out 6^th^ January 2015; retrieved 6323 records  EMBASE 8^th^ January 2015; retrieved 9296 records  CINHAL 13^th^ January 2015; retrieved 1977 records  COCHRANE (CENTRAL) 26^th^ January 2015 5853 records  After deduplication:  MEDLINE = 4813  EMBASE = 5203  CINAHL = 1824  COCHRANE (CENTRAL) = 5095 |
| --- |
